# The dsRNA Virus Papaya Meleira Virus and an ssRNA Virus Are Associated with Papaya Sticky Disease

**DOI:** 10.1371/journal.pone.0155240

**Published:** 2016-05-11

**Authors:** Tathiana Ferreira Sá Antunes, Raquel J. Vionette Amaral, José Aires Ventura, Marcio Tadeu Godinho, Josiane G. Amaral, Flávia O. Souza, Poliane Alfenas Zerbini, Francisco Murilo Zerbini, Patricia Machado Bueno Fernandes

**Affiliations:** 1 Núcleo de Biotecnologia, Universidade Federal do Espírito Santo, Vitória, Espírito Santo, Brazil; 2 Instituto Capixaba de Pesquisa, Assistência Técnica e Extensão Rural, Vitória, Espírito Santo, Brazil; 3 Dep. de Fitopatologia/BIOAGRO, Universidade Federal de Viçosa, 36570–900, Viçosa, Minas Gerais, Brazil; 4 Dep. de Microbiologia/BIOAGRO, Universidade Federal de Viçosa, 36570–900, Viçosa, Minas Gerais, Brazil; Universidade Federal do Rio Grande do Sul, BRAZIL

## Abstract

Papaya sticky disease, or “meleira”, is one of the major diseases of papaya in Brazil and Mexico, capable of causing complete crop loss. The causal agent of sticky disease was identified as an isometric virus with a double stranded RNA (dsRNA) genome, named papaya meleira virus (PMeV). In the present study, PMeV dsRNA and a second RNA band of approximately 4.5 kb, both isolated from latex of papaya plants with severe symptoms of sticky disease, were deep-sequenced. The nearly complete sequence obtained for PMeV dsRNA is 8,814 nucleotides long and contains two putative ORFs; the predicted ORF1 and ORF2 display similarity to capsid proteins and RdRp's, respectively, from mycoviruses tentatively classified in the family *Totiviridae*. The sequence obtained for the second RNA is 4,515 nucleotides long and contains two putative ORFs. The predicted ORFs 1 and 2 display 48% and 73% sequence identity, respectively, with the corresponding proteins of papaya virus Q, an umbravirus recently described infecting papaya in Ecuador. Viral purification in a sucrose gradient allowed separation of particles containing each RNA. Mass spectrometry analysis indicated that both PMeV and the second RNA virus (named papaya meleira virus 2, PMeV2) were encapsidated in particles formed by the protein encoded by PMeV ORF1. The presence of both PMeV and PMeV2 was confirmed in field plants showing typical symptoms of sticky disease. Interestingly, PMeV was detected alone in asymptomatic plants. Together, our results indicate that sticky disease is associated with double infection by PMeV and PMeV2.

## Introduction

Papaya sticky disease ("meleira" in Portuguese) was first reported in 1980 in the south of Bahia and north of Espírito Santo states of Brazil [1]. The disease has spread and currently occurs also in the states of Pernambuco, Ceará, Rio Grande do Norte and Minas Gerais [2]. Besides Brazil, the disease has been confirmed only in Mexico [3, 4].

Papaya sticky diseased fruits and leaves present an unprompted/natural exudation of fluid latex. The oxidation of the latex after atmospheric exposure leads to necrotic lesions on young leaves edges, and also gives a sticky aspect to the fruit, rendering them unacceptable for commercial consumption [5]. During the disease, potassium levels and the osmotic balance in lactiferous vessels are modified, leading to cell rupture and latex exudation [6]. Some plants also display necrosis of the leaf tips, although this symptom is not always associated with the disease.

To date, the best available strategy to control the disease is the roguing of infected plants. However, since the symptoms become apparent only after fruit setting, an infected symptomless plant in the field may remain unnoticed for a long period, acting as a virus source [4]. For this reason, efforts have been focused on the characterization of the causal agent(s) and the development of diagnostic tools for early detection.

A viral etiology of sticky disease was suggested by Kitajima et al, [7] following the observation of dsRNA-containing isometric particles in the laticiferous vessels of infected plants. Maciel‐Zambolim et al, [8] expanded these results with the detection of a double-stranded RNA (dsRNA) virus, named papaya meleira virus (PMeV), in diseased papaya plants, and the purification of isometric particles of approximately 42 nm in diameter from papaya latex. PMeV dsRNA was estimated to be either ~10 kb [7] or ~12 kb [8] in length by agarose gel electrophoresis. The viral etiology was confirmed after healthy papaya plants inoculated with purified virus particles developed typical symptoms of the sticky disease [8].

Comparative analysis of a ~560 bp fragment, corresponding to the RNA-dependent RNA polymerase (RdRp) gene, amplified from PMeV isolates collected in the major Brazilian papaya-producing states, suggested that PMeV possesses a similarity with mycoviruses of the family *Totiviridae* [2, 9]. The complete sequence of a PMeV isolate collected at Rio Grande do Norte state, Brazil, was reported by Abreu et al, [10]. *In silico* analysis of the 8.7 kb genome revealed that the deduced amino acid sequence of PMeV ORF2 contains the conserved domains characteristic of RdRps from members of the genera *Luteovirus*, *Totivirus* and *Rotavirus*. Until now, PMeV remains unclassified by the International Committee on Taxonomy of Viruses (ICTV).

In Mexico, symptoms similar to those of sticky disease have been observed in papaya cv. Maradol. Curiously, although latex exudation has been reported to be more severe than in Brazil, necrosis of the leaf tips has not been observed. The presence of a viral dsRNA in diseased plants was confirmed, and it was possible to transmit the disease through the latex of infected plants to healthy plants [3] and also by seeds in cv. Maradol [11]. However, a cDNA library generated from papaya latex infected by a Mexican isolate (named PMeV-Mx) identified a 1154-bp sequence encoding a putative RdRp showing closer similarity to umbraviruses and no similarity to PMeV-RN. Nevertheless, primers designed based on the PMeV-Mx sequence amplified fragments from both Brazilian and Mexican infected plants, and the amplified sequences had 100% identity at the nucleotide level [12].

It is unlikely that papaya sticky disease in Brazil and in Mexico, displaying similar symptoms, would have different viruses as causal agents. Thus, we hypothesized that PMeV and a second, umbra-like virus, could be involved in the disease. To test this hypothesis, we deep-sequenced RNA purified directly from latex samples, analyzed the association of different RNA molecules with the isometric particles found in the laticiferous vessels, and tested for the presence of these RNA molecules in symptomatic and symptomless field plants. Our results are consistent with the presence of two RNA molecules with different sizes and genomic organization in the latex of infected plants. Thus, a new etiology is proposed for papaya sticky disease: it is associated with a double infection with two viruses, PMeV and an ssRNA virus which is closely related to members of the genus *Umbravirus*.

## Methods and Materials

### Ethics Statement

Specific authorization is not required for this experiment since the author and coauthors are Professors and researchers in the Federal University of Espirito Santo (UFES) and Capixaba Institute of Research, Technical Assistance and Rural Extension (Incaper). Incaper and UFES have an official cooperation agreement (Prot. 70557870) for agricultural and biological research. Samples were obtained from plants cultivated at Incaper Experimental Farms in the north and southwest of Espirito Santo state.

### Plant material

For deep-sequencing and virus purification, latex samples of papaya plants with typical symptoms of sticky disease were collected at Sooretama, Espírito Santo (ES) state, Brazil. Latex was sampled from each plant using a sterile scalpel blade and mixed (1:1, v/v) with 0.1 M sodium citrate buffer pH 5.0 [13].

Additional samples were collected for detection of sticky disease-associated viruses. Latex (5 ml) and leaf (~2 g) samples of papaya cv. Golden were collected at Baixo Guandu, Iuna, Sooretama and Vitória, ES. Samples were divided into two categories: (1) 55 symptomatic plants for sticky disease and (2) 42 asymptomatic plants. Latex samples were mixed (1:1, v/v) with 0.1 M sodium citrate buffer pH 5.0, cooled in ice and stored at -20°C; leaf samples were cooled in dry ice and stored at -80°C.

### Viral genome sequencing and assembly

Viral RNAs were extracted from latex of infected plants as described by Rodrigues et al, [13]. Viral RNA bands were excised from agarose gels and purified using the PureLink Quick Gel Extraction Kit (Invitrogen). The purity was assessed based on the A260/A280 ratio with a Nanodrop ND1000 spectrophotometer (Thermo Scientific).

Viral RNAs were sent to Macrogen Inc. (Seoul, South Korea) for deep-sequencing using the 454 GS-FLX Titanium platform. The reads were trimmed and quality-filtered. *De novo* assembly of the viral genome was carried out using the GS *De Novo* Assembler (v. 2.6) and SeqMan NGen (DNAStar) with default parameters. The assembled contigs were compared against sequences in GenBank using the BLASTX algorithm.

To confirm the authenticity of the 454-derived sequence, specific primers (S1 Table) were designed for genome amplification and Sanger sequencing. RNA extracted from purified virions as described by Maciel-Zambolim et al, [8] was used as a template for genome amplification. RNA was incubated at 96°C for 3 min or 70°C for 10 min to denature viral dsRNA and viral ssRNA genomes, respectively. First-strand cDNA synthesis was performed using random hexamers and Superscript III Reverse Transcriptase (Invitrogen) following the manufacturer's instructions. All PCR reactions used Platinum High Fidelity DNA Polymerase (Invitrogen) following the manufacturer's instructions in a Mastercycler Thermocycler (Eppendorf). Reactions were performed using the following program: 94°C for 3 min followed by 35 cycles of 94°C for 45 s, Tm* for 30 s (S1 Table), 72°C for 2.5 or 1 min (for ~2000 bp and ~1000 bp, respectively), and a 10-min final extension at 72°C. The amplicons were sent to Macrogen for Sanger sequencing.

### Sequence and phylogenetic analyses

Viral consensus sequences of approximately 9 kb for PMeV and 4.5 kb for the second virus (PMeV2) were scanned for the presence of ORFs using Genemark (http://exon.gatech.edu/GeneMark/eukhmm.cgi). Prediction of pseudoknot structures was performed with DotKnot [14] using as input the 100-nt sequence immediately downstream of the putative heptanucleotide at the ORF1-ORF2 junction of the PMeV coding strand.

Sequence identity/similarity for each ORF was determined using BLAST (http://blast.ncbi.nlm.nih.gov). MUSCLE [15] was used to align RdRp amino acid sequences and the conserved domain RdRP_4 (pfam02123) from PMeV, 14 members of the family *Totiviridae* and 10 toti-like viruses, and to align RdRp amino acid sequences and the conserved domain RdRP_3 (pfam00998) from PMeV2, 16 members of the family *Tombusviridae* and three umbra-like viruses (S2 Table). Phylogenetic trees were constructed using maximum likelihood and Bayesian inference. Maximum likelihood trees were constructed using MEGA6 (substitution model WAG+G+I) and tested with a bootstrap of 10,000 replicates to ascertain the reliability of each branch pattern. Bayesian inference was performed with MrBayes v. 3.0b4 [16], with the model selected by MrModeltest v. 2.2 (Nylander, 2004) in the Akaike Information Criterion. The analyses were carried out running 20,000,000 generations, excluding the first 2,000,000 generations as burn-in. Trees were visualized using Fig Tree (http://tree.bio.ed.ac.uk/software/figtree/).

### Virus purification and characterization of viral structural proteins

Latex collected from diseased papaya plants was centrifuged through a 10–40% (w/v) linear sucrose gradient for virus purification as described by Maciel-Zambolim et al, [8]. Final pellets were resuspended in tris-borate buffer pH 9.0 and the purified virus was stored at 4°C. The virus suspensions were scanned with UV light in the 220–320 nm range to assess their purity. Virus particles from these preparations were negatively stained in 2% potassium phosphotungstate, pH 6.8, for examination in a Zeiss EM-109 transmission electron microscope (TEM).

To identify the structural proteins, purified viral particles collected the from M fraction were digested with trypsin according to Shevchenko et al, [17]. Tryptic peptides were analyzed by MALDI-TOF/TOF (Bruker Daltonics Ultraflex III) and sequenced peptides were compared against the predicted proteins in the viral genome using Prodigal software (downloaded on 01.07.2015, with 12 entries), using the MASCOT application version 2.4.0 (Matrix Science, London, UK). The results obtained by MASCOT were validated by SCAFFOLD version 3.6.4 (Proteome Software Inc., Portland, OR), using the Peptide Prophet [18] and Protein Prophet [19] algorithms with acceptance criteria of 99.9% probability of identification for proteins and 95% for peptides. Putative protease cleavage sites on putative proteins were predicted using NetPicoRNA 1.0 Server [20].

### Viruses detection by conventional RT-PCR

Viral RNAs were extracted from latex samples using Trizol reagent (Ambion), according to the manufacturer instructions. The final RNA pellet was dissolved in 20 μL of nuclease-free water and stored at -80°C. The purity (A_260_/A_280_) was assessed with a Nanodrop ND1000 spectrophotometer. The purified RNA (1 μg) was treated with DNAse I (Invitrogen) and incubated at 96°C for 3 min or 70°C for 10 min to denature dsRNA and ssRNA, respectively. First-strand cDNA synthesis was performed using random hexamers and M-MLV Reverse Transcriptase (Invitrogen) following the manufacturer instructions. All PCR reactions used recombinant Taq DNA Polymerase (Invitrogen) in a Mastercycler Thermocycler (Eppendorf). Reactions were performed using the following programs: (i) PMeV primers: 94°C for 3 min followed by 35 cycles of 94°C for 45 s, 62°C for 30 s, 72°C for 1 min, and a 10-min final extension at 72°C; (ii) PMeV2 primers: 94°C for 3 min followed by 35 cycles of 94°C for 45 s, 62°C for 30 s, 72°C for 1 min, and a 10-min final extension at 72°C; (iii) Luteovirus primers: 94°C for 3 min followed by 35 cycles of 94°C for 45 s, 57°C for 45 s, 72°C for 1 min, and a 10-min final extension at 72°C. PCR amplicons were visualized on 1% agarose gels.

## Results

### Virus identification and genome sequencing

Viral nucleic acids were extracted from latex samples of infected plants (cv. Golden) and two bands could be visualized in agarose gels, one of approximately 10 kb, likely corresponding to the PMeV dsRNA (henceforth named PMeV-ES), and a second band of approximately 4.5 kb (S1 Fig). Both RNA bands were sliced out, purified from the agarose gel and sent to Macrogen, Inc. for deep-sequencing.

The data obtained from RNA sequencing was comprised of 54,602 reads and 22,706,988 nt, with an average read length of 415 nt. Following *de novo* assembly, the contigs were submitted to a BLASTX search. The results indicated that it was possible to separate the contigs into two groups, each showing identity to different known viral RNA sequences. One group showed the lowest e-values with the putative structural protein of PMeV-RN (KT013296), and with the RdRp) protein of *Phlebiopsis gigantea mycovirus dsRNA-2* (PgV-2; AM111097), a dsRNA virus related to members of family *Totiviridae*. The other group showed the lowest e-values with the RdRp of papaya virus Q (PpVQ; KP165407), a new papaya virus discovered in Ecuador and which, like PMeV-Mx, is related to members of the genus *Umbravirus*. Therefore, the 4.5 kb band is not a viral subgenomic RNA as previously suggested by Kitajima et al, [7] and Maciel-Zambolim et al, [8]. Instead, it corresponds to a second virus which is related to umbraviruses. The authenticity of the deep-sequencing data was validated by Sanger sequencing.

### Genome organization and characteristics of the two viruses associated with papaya sticky disease

The sequence obtained for PMeV-ES is 8,814 nucleotides long (GenBank accession no. KT921784). Previous studies had estimated the length of the PMeV dsRNA to be 10–12 kb by agarose gel electrophoresis [7, 8]. However, as pointed out by Abreu et al, [10], these studies used a dsDNA ladder for the determination of dsRNA length. The electrophoretic mobility of dsRNA is lower than that of dsDNA [21], which may explain the difference in size of the PMeV dsRNA observed in those earlier studies and the genome sequence obtained here and also by Abreu et al, [10].

**Fig 1:**
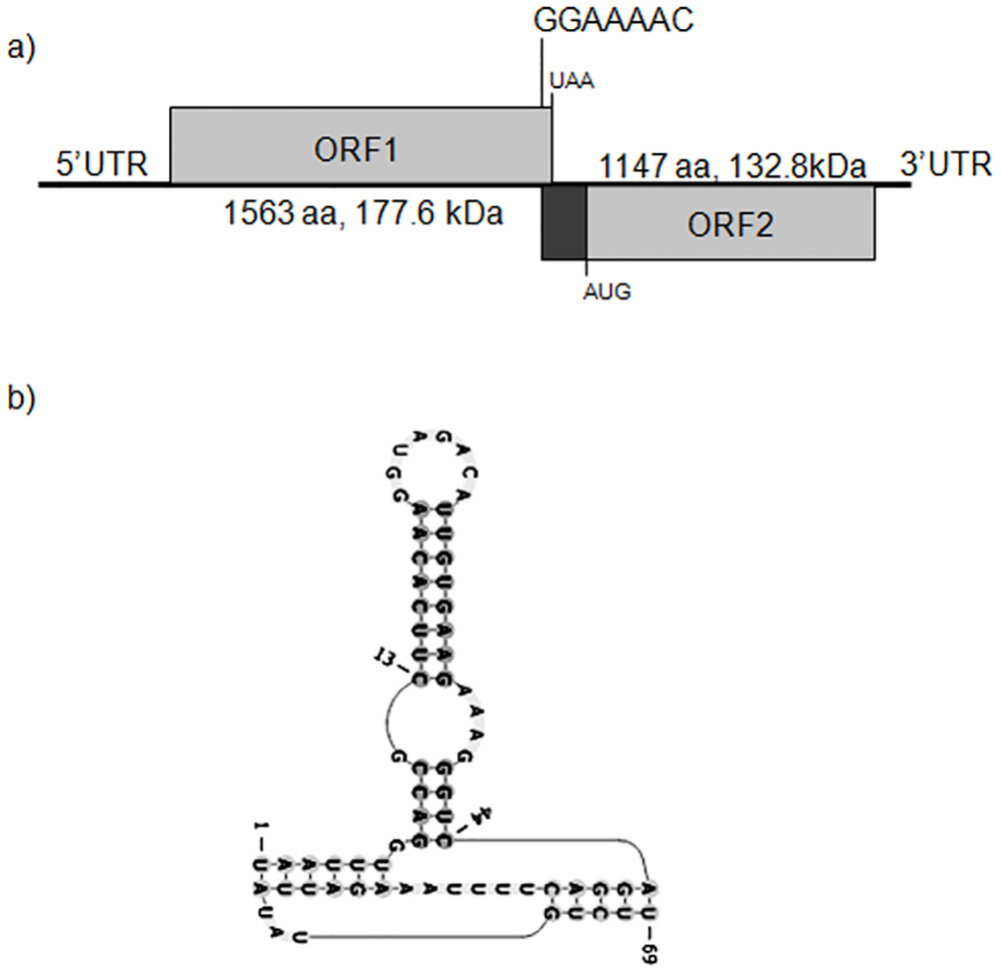
Genome organization and molecular features of papaya meleira virus (PMeV). (a) Schematic representation of the PMeV genomic organization. The viral dsRNA contains two ORFs. ORF1 encodes the putative coat protein (CP) and ORF2 encodes a putative RNA-dependent RNA polymerase (RdRp). The position of a putative slippery sequence for -1 translational frameshift is indicated at the 3'-end of ORF1. (b) Predicted pseudoknot structure located downstream of the slippery sequence.

PMeV has two putative ORFs in different reading frames (Fig 1). The predicted ORF1 (nt 615–5306) encodes a putative polypeptide of 1,563 aminoacids with a predicted molecular mass of 177.6 kDa. This polypeptide is 75% identical to the one from PMeV-RN and 20–26% identical to the analogous proteins from the mycoviruses *Sclerotinia sclerotiorum nonsegmented virus-L* (SsNsV-L; NC_017915), *Botrytis cinerea RNA virus-1* (BcRV-1; NC_026139), *Fusarium virguliforme dsRNA mycovirus-1* (FvRV-1; JN671444; and *Fusarium graminearum dsRNA mycovirus-3* (FgV-3; NC_013469). Strikingly, no significant matches are found for any of these putative proteins in protein databases. These currently unclassified viruses are related to viruses in the family *Totiviridae*, and contain two ORFs in an arrangement which is characteristic of this family (Fig 1a). ORF1 is speculated to be a structural/gag protein, even though various purification protocols failed to yield particles of *Phytophthora infestans* RNA virus 3 (PiRV-3) [22] and PgV-2 [23].

Totiviruses typically contain two open reading frames (ORFs) that can be translated as a fusion protein by a -1 frameshift. Sequence analysis of PMeV-ES identified a potential slippery heptamer, GGAAAAC, at nt 5,297–5,303, immediately before the ORF1 stop codon (Fig 1a). The same putative frameshifting sequence is present in PMeV-RN, and similar structures have been identified in the five viruses most closely related to PMeV (PgV-2, FgV-3, PiRV-3, SsNsV-L and BcRV-1). In these viruses, the slippery sequences differ from the general heptanucleotide XXXYYYZ, where X represents any nucleotide, Y represents A or U, and Z represents A, C or U [22, 24, 25]. Following the slippery sequence there is a stable secondary structure, such as a pseudoknot or hairpin [26]. PMeV-ES has a predicted RNA pseudoknot structure that lies closely downstream of the ORF1 stop codon (Fig 1b).

**Fig 2:**
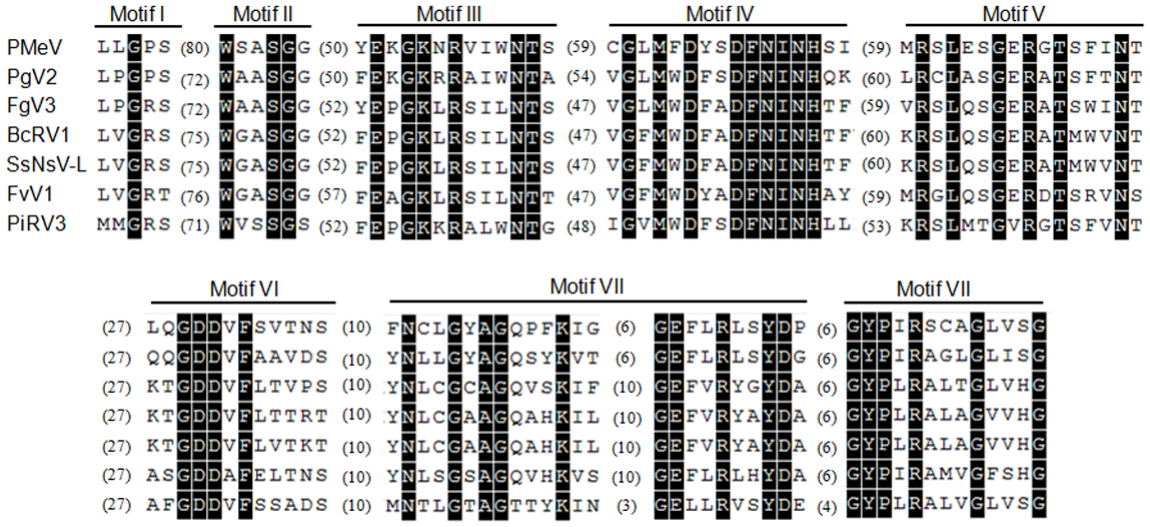
Alignment of the RdRp4 domain from PMeV and related viruses. Alignment of the RdRp4 domain (pfam02123) identified in ORF2 of papaya meleira virus (PMeV) with the corresponding regions of the most closely related totivirid-like viruses (see S2 Table for full virus names and GenBank access numbers). Sequences were aligned with MUSCLE (Edgar, 2004). The numbers in parenthesis indicate the number of amino acid residues separating individual motifs. Amino acid residues highlighted in black are conserved among all aligned sequences.

The predicted ORF2 (nt 5366–8809) encodes a putative protein of 1,147 aminoacids with a predicted molecular mass of 132.8 kDa and 67% identity with the ORF2 protein from PMeV-RN. This protein contains the conserved domain of RdRp superfamily 4 (pfam02123: RdRP_4), which includes the RdRps of luteoviruses, rotaviruses and totiviruses. The PMeV RdRp has eight motifs (I to VIII) (Fig 2), which are conserved in the RdRps in BcRV-1, FgV-3, FvRV-1, PgV-2, PiRV-3 and SsNsV-L. It has 32% identity with the RdRp's of PgV-2 and FgV-3, 31% identity with the RdRp's of BcRV-1and SsNsV-L, and 29% identity with the RdRp of FvRV-1. Many other members of the family *Totiviridae* showed similarity to PMeV RdRp but with lower coverage and identity (data not shown).

**Fig 3:**
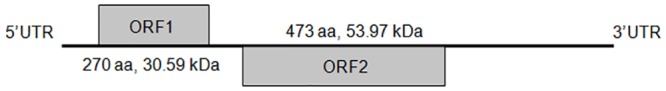
Schematic representation of the genomic organization of papaya umbraviruspapaya meleira virus 2 (PpUVPMeV2). The viral ssRNA contains two ORFs. ORF1 encodes a hypothetical protein and ORF2 encodes a putative RdRp.

The partial sequence obtained for the umbravirus-like RNA is 4,515 nucleotides long (GenBank accession no. KT921785), and contains two putative ORFs in different reading frames (Fig 3). The nucleotide sequence has 70% and 71% identity with PpVQ and PMeV-Mx, respectively (the sequences of PpVQ and PMeV-Mx are 79% identical).

The predicted ORF1 (nt 4–816) encodes a putative polypeptide of 270 aminoacids with a predicted molecular mass of approximately 31kDa. This polypeptide has 48% identity to the corresponding proteins of both PMeV-Mx and PpVQ. No significant matches are found for any of these putative proteins in protein databases.

The predicted ORF2 (nt 978–2399) encodes a putative protein of 473 aminoacids with a predicted molecular mass of 53.97 kDa. This protein contains the conserved domain of RdRp superfamily 3 (pfam00998: RdRP_3), which includes RdRp's of various plant viruses. It has 73% identity to the RdRp of PpVQ, 66% identity to the RdRp of PMeV-Mx, 43% identity to the RdRp of the unclassified *Citrus yellow vein-associated virus* (CYVaV; JX101610), 43% identity to the RdRp of the umbravirus *Carrot mottle mimic virus* (CMoMV; NC_001726) and 40% identity to the RdRp of the umbravirus *Carrot mottle virus* (CMoV; NC_011515). Many other members of the genus *Umbravirus* also show similarity (data not shown).

Together, these sequence comparison results indicate that the 4.5 kb RNA detected in papaya plants with sticky disease corresponds to an umbravirus which is closely related to both PMeV-Mx and PpVQ. However, considering that the complete sequences of PMeV-Mx and PpVQ have not yet been determined and that the nucleotide sequence identities between the currently available sequences (70–79%) are near the threshold for species demarcation in the genus *Umbravirus* [27], it is not possible to state at this time whether these are isolates of the same species, or distinct species. Thus, we will refer to the second RNA virus isolated from papaya plants with sticky disease as papaya meleira virus 2 (PMeV2-ES). Determination of the complete sequences of these three isolates will be necessary to clarify their taxonomic status.

### Phylogenetic analyses

**Fig 4:**
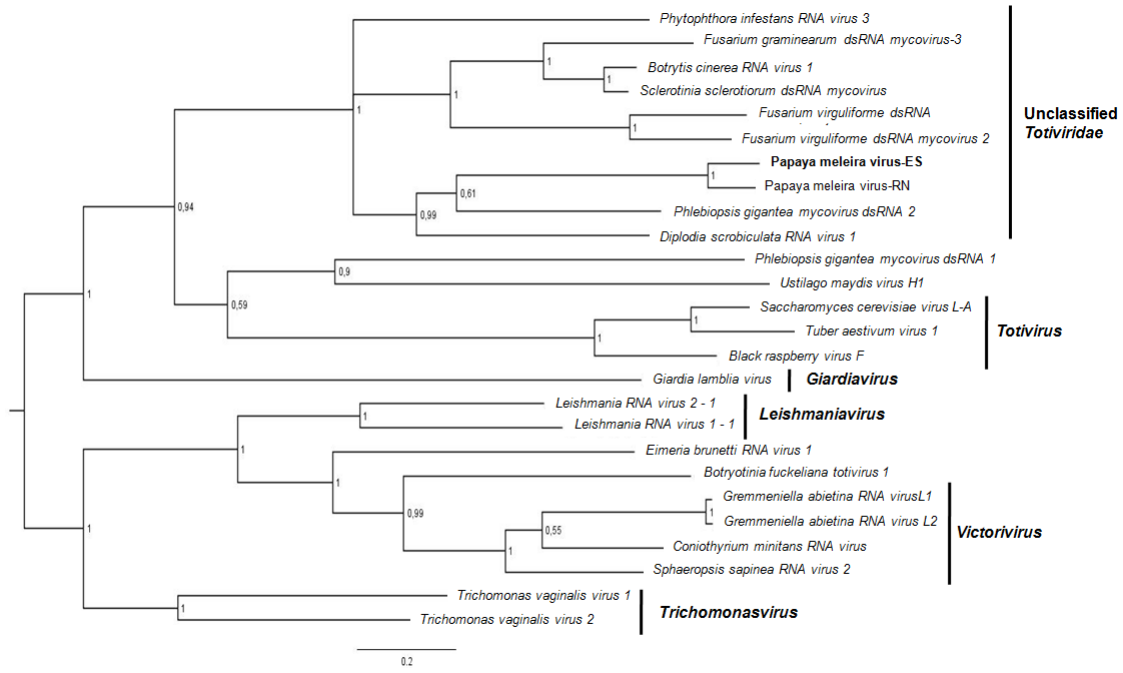
Phylogenetic relationship between PMeV and related viruses. Phylogenetic tree based on an alignment of deduced amino acid sequences of the RdRp proteins from PMeV and members of the family *Totiviridae*, obtained using Bayesian inference. The numbers at the branch nodes indicate Bayesian posterior probabilities. The names of the viruses used in the analysis and their respective GenBank access numbers are listed in S2 Table.

Phylogenetic trees based on RdRp deduced amino acid sequences were constructed to evaluate the evolutionary relationship between PMeV, 14 members of the family *Totiviridae*, and 10 toti-like viruses (S2 Table). Unassigned totivirid-like viruses formed a strongly supported clade, distinguishable from all other viruses in the family (Fig 4). Two main groups can be recognized within this cluster, one cluster corresponding to PMeV, PiRV-3, PgV-2, DsRV-1, and the other to FvRV-1, FvRV-2, BcRV-1, SsNsV-L and FgV-3. These viruses appear to be more closely related to fungal viruses in the genus *Totivirus* than to other protozoan and fungal viruses in the genera *Giardiavirus*, *Victorivirus*, *Trichomonasvirus* and *Leishmaniavirus*.

**Fig 5:**
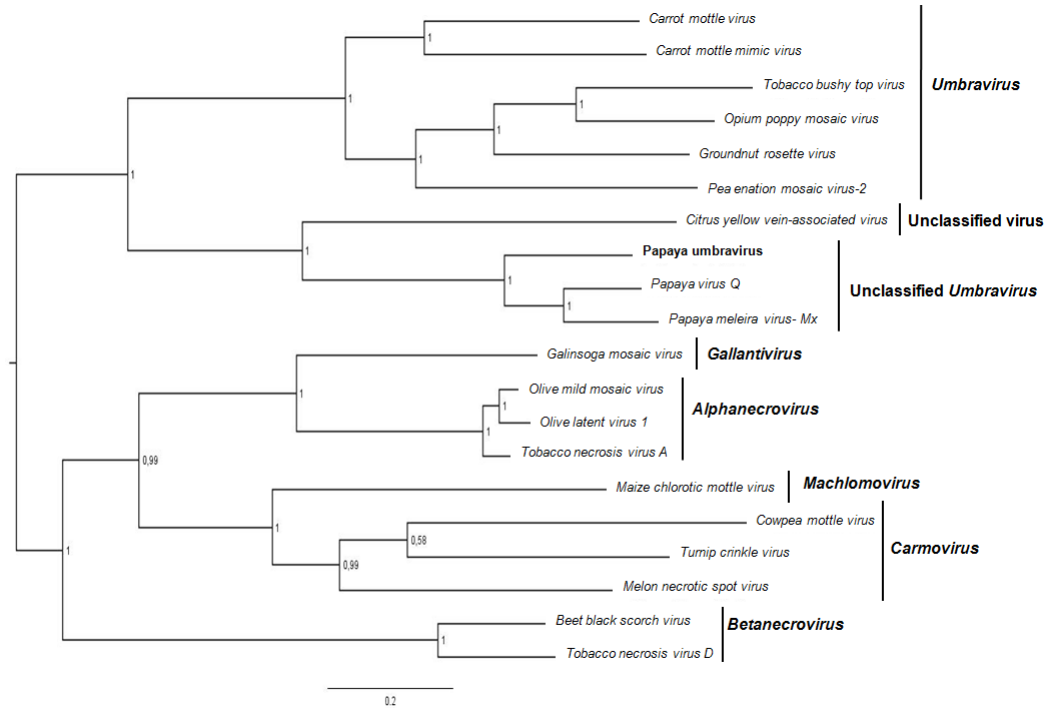
Phylogenetic relationship between PpUVPMeV2 and related viruses. Phylogenetic tree based on an alignment of the deduced amino acid sequences of PpUVPMeV2 and members of the family *Tombusviridae*, obtained using Bayesian inference. The numbers at the branch nodes indicate Bayesian posterior probabilities. The names of the viruses used in the analysis and their respective GenBank access numbers are listed in S2 Table.

Phylogenetic trees based on RdRp deduced amino acid sequences were also constructed to evaluate the evolutionary relationship between PMeV2-ES, PpVQ, PMeV-Mx and 16 members of the family *Tombusviridae* (S2 Table). The midpoint-rooted tree showed two main branches: one formed by PMeV2-ES, CYVaV, PpVQ, PMeV-Mx and members of the genus *Umbravirus*, and the other formed by viruses in the genera *Gallantivirus*, *Alphanecrovirus*, *Machlomovirus*, *Carmovirus* and *Betanecrovirus*. Interestingly, PMeV2, PpVQ and PMeV-Mx formed a clade distinct from all the other viruses with a high statistical support (Fig 5). These clusters were also observed in a maximum likelihood tree (data not shown), indicating that they reflect true evolutionary relationships, regardless of the method used for phylogenetic reconstruction.

### Virus purification and characterization of viral RNA and structural proteins

**Fig 6:**
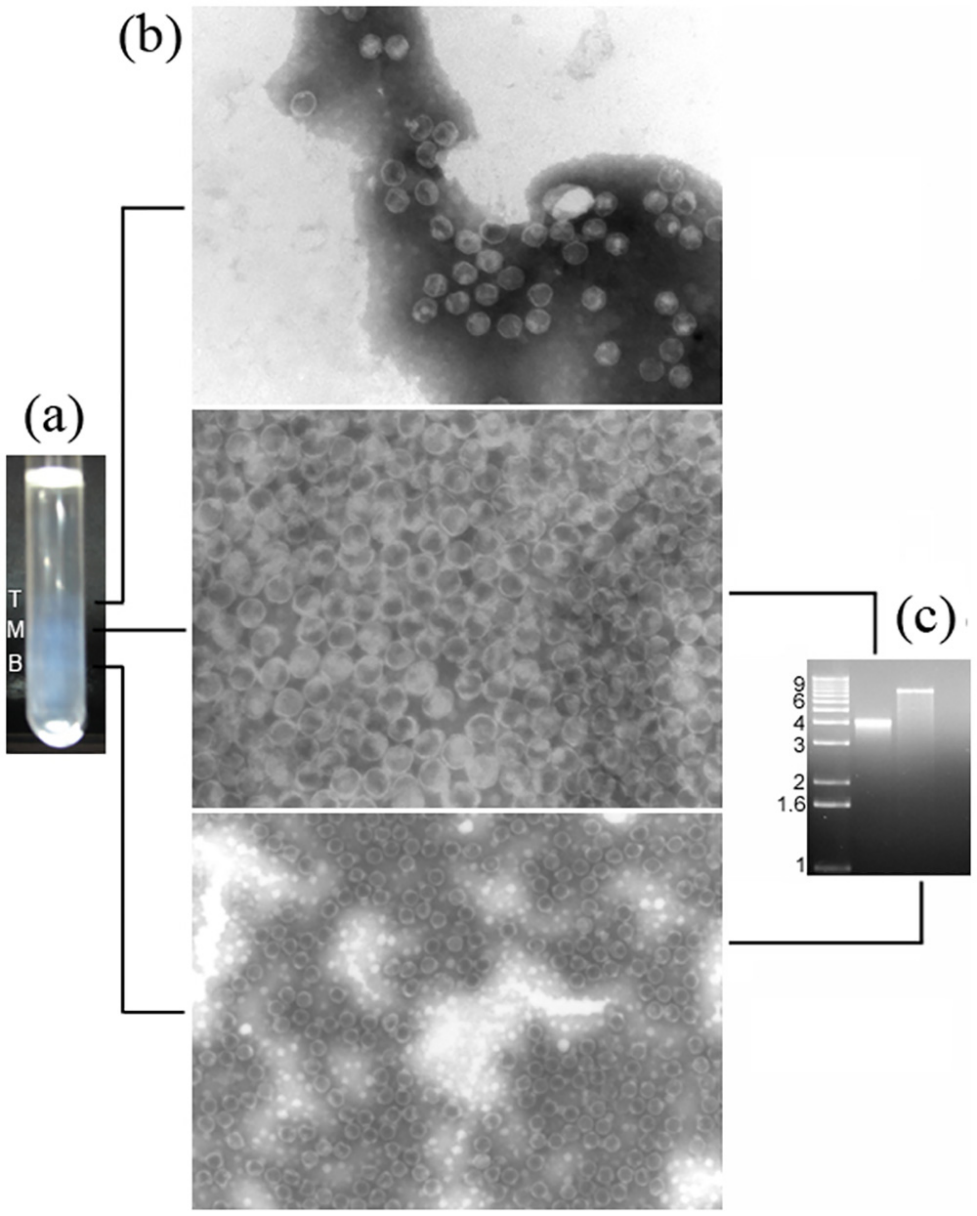
Analysis of purified viral preparations from the latex of papaya plants displaying severe symptoms of sticky disease. (a) Viral bands after centrifugation in a sucrose density gradient. T, top; M, medium. B, bottom. (b) Transmission electron microscope images of viral particles from the T, M and B fractions, as indicated. Images T and M, 140,000x. Image B, 85,000x. (c) Agarose gel electrophoresis of RNA extracted from particles in the M and B fractions, as indicated. No RNAs were obtained from particles in the T fraction. The left lane is a size marker [1 kb plus DNA ladder (Invitrogen), in kbp].

Previous electron microscopy studies have indicated the presence of a single type of viral particles (isometric, 42 nm in diameter) in papaya plants with sticky disease. To confirm the association of two distinct genomic RNAs with these particles, virions were purified in a sucrose density gradient. Careful observation of the gradient indicated the presence of three well-defined opalescent zones (Fig 6a). The three fractions were carefully collected and visualized separately by TEM (Fig 6b). Interestingly, the same particle size and morphology was observed for all fractions; however, when purified RNAs from each fraction were subjected to denaturing electrophoreses, a ~4,5 kb band was visualized from the middle fraction, and a ~9 kb band from bottom fraction (Fig 6c); no RNA bands were visualized from the top fraction.

**Fig 7:**
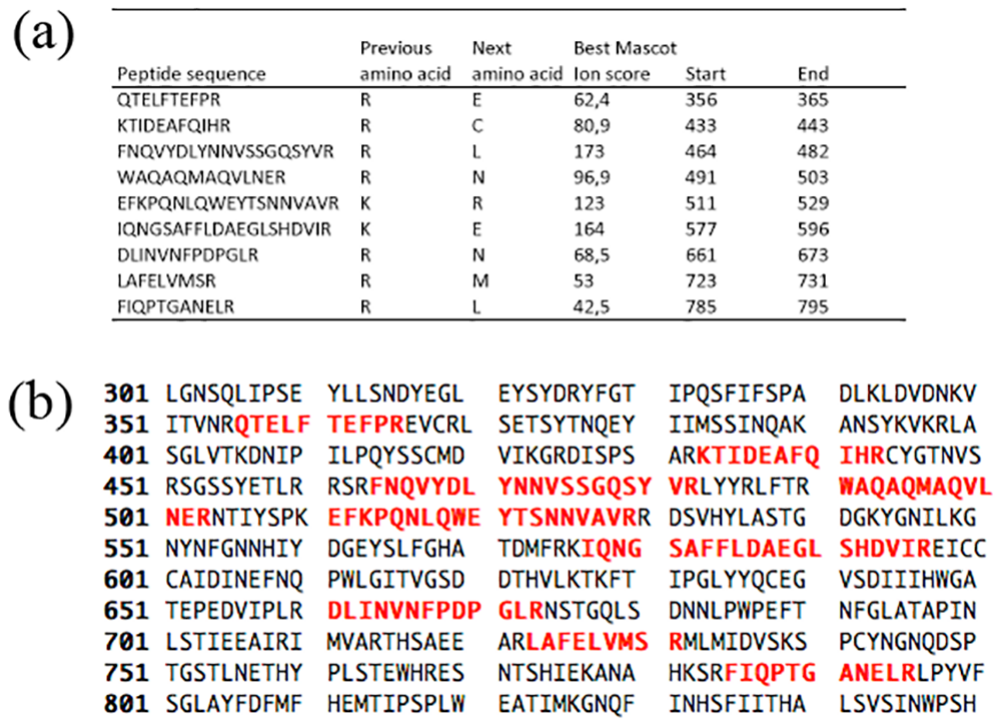
Peptide mapping of the PMeV/PpUVPMeV2 structural (CP) protein in the deduced amino acid (aa) sequence of PMeV ORF1. (a) List of the nine peptides identified by mass spectrometry, including their MASCOT scores and their coordinates (start and end) within the ORF1 aa sequence. (b) Deduced amino acid sequence of the central portion of ORF1, highlighting the positions of the nine peptides listed in (a). Note that all nine peptides display 100% identity with the ORF1 aa sequence. The complete amino acid sequence of PeMV ORF1 is 1563 aa long.

Viral structural proteins from purified virions collected from the M fraction were identified by mass spectrometry. A total of nine peptide fragments matched the PMeV ORF1-encoded polypeptide sequence (Fig 7), accounting for 8% of the amino acid sequence (125/1563 aa) and encompassing the central region of the putative protein (from aa 356 to 785). This result indicated that the PMeV ORF1 translated product could either undergo self-cleavage or be cleaved by papaya latex proteases, and that the cleaved product(s) comprise(s) the viral structural proteins.

NetPicoRNA 1.0 server was used to predict cleavage sites at positions 313 and 1427 of the deduced aa sequence of PMeV ORF1 would result in a mature CP with a calculated molecular mass of approximately 122 kDa. However, viral capsid proteins of non-enveloped, isometric viruses are usually smaller (17–40 kDa) [28]. Additional cleavage sites with atypical consensus sequence were not detected by the algorithms employed.

### Papaya sticky disease is associated with the presence of PMeV and PMeV2

To verify whether papaya sticky disease is indeed associated with double infection by PMeV and PMeV2, 55 latex samples of symptomatic plants and 42 latex samples of asymptomatic plants were collected and used for virus detection by RT-PCR. The presence of both PMeV and PMeV2 was confirmed for all symptomatic plants. In the 42 asymptomatic plants, PMeV alone was detected in 33 plants, and 9 plants had the two viruses. Asymptomatic plants infected by PMeV and PMeV2 were monitored for at least 3 months after fructification and, in some cases, eventually developed typical symptoms, while plants infected only by PMeV remained asymptomatic.

## Discussion

Initial studies analyzing plants with typical symptoms of sticky disease were based upon the extraction of dsRNA from the plant latex using organic solvents, followed by gel electrophoresis to visualize the resultant dsRNA bands [7, 8, 13]. Usually two bands could be visualized, one at ~10 kb likely corresponding to PMeV dsRNA, and a second band of approximately 4.5 kb. Sequences of the larger RNA indicated a similarity with fungal viruses related to the family *Totiviridae* [2, 9, 10]. The smaller band was regarded as a viral subgenomic RNA. However, following recent reports of an umbravirus-related virus associated with sticky disease in Mexico, we decided to sequence and characterize both RNA molecules.

Extended sequence obtained from the 9 kb RNA (PMeV-ES), indicates that it contains two putative ORFs in an arrangement characteristic of members of the family *Totiviridae*. This family includes viruses of fungi and protozoa that form virions (*ie*, they encode a coat protein which encapsidates the viral genome) and have a single-component dsRNA genome [29].

The majority of known plant viruses have RNA genomes that generate double-stranded RNA (dsRNA) replicative forms at some point of their life cycle. Thus, dsRNA has been used to assess RNA virus infection in plants. On this regard, biodiversity surveys of plant viruses obtained from dsRNA extraction and sequencing have been done and, surprisingly, members of the *Totiviridae* family have been identified [30]. Liu et al, [31] performing *in silico* cloning using known dsRNA viral sequences as queries to search against the NCBI Expressed Sequence Tag (EST) database, obtained plant virus-like sequences that are related to members of the family *Totiviridae*. In another approach, small RNA (smRNA) sequencing of maize plants from southwest China resulted in the identification of a new dsRNA virus with a sequence and genomic organization resembling that of a totivirus [32]. Interestingly, siRNA sequencing from *C*. *papaya* identified totivirus-like sequences [33].

The sequence obtained from the smaller, 4.5 kb RNA, corresponds to a virus related to species in the genus *Umbravirus*, here named PMeV2-ES. PMeV2-ES has high similarity to PMeV-Mx and to PpVQ, a new umbra-like virus recently described in Ecuador [34]. PMeV-Mx was detected in papaya plants showing typical symptoms of sticky disease in Mexico [12]. PpVQ was detected in papaya plants also infected by *Papaya ringspot virus* (PRSV). It is noteworthy that symptoms of sticky disease have not been reported in Ecuador. Sequence similarity and phylogenetic analysis including PMeV2, PpVQ and PMeV-Mx suggest that they may be different isolates of the same umbravirus, or closely related umbraviruses. Either way, they are distinct from the dsRNA-containing PMeV.

Umbraviruses are distinguished from most other viruses for their lack of a coat protein (CP) gene and, as a result, umbraviruses do not form conventional virus particles. In nature, they are dependent on the presence of a helper virus—typically, a member of the family *Luteoviridae*. The CP of the helper virus forms hybrid virus particles encapsidating the umbraviral RNA, which can be transmitted by the helper virus vector to other plants [35]. Since our protein sequence data provided no indication of the presence of a luteovirus in infected papaya plants and no luteovirus was detected in papaya sticky diseased plants using universal luteovirus primers [36], we assumed that PMeV is the helper virus of PMeV2. To identify the capsid protein responsible for the encapsidation of PMeV2 RNA, the structural protein(s) from purified virions were analyzed by mass spectrometry. All sequenced peptides match with the deduced amino acid sequence of ORF1 from PMeV, strongly indicating that PMeV acts as the helper virus for PMeV2 and that the PMeV CP forms hybrid virus particles encapsidating PMeV2 ssRNA. To the best of our knowledge, this is the first known example of a viral coat protein being capable of encapsidating both ssRNA and dsRNA forms, and also the first known case of an umbravirus associated with a non-luteovirid.

Interestingly, all papaya plants analyzed, symptomatic or asymptomatic, were infected by PMeV, suggesting that this virus alone cannot induce sticky disease symptoms. Persistent plant viruses are associated with latent infections and have little or no overt effects on their hosts. They are found in crops, including beans, peppers and rice, where they have been studied more extensively [37]. The origin of persistent plant viruses is unknown, but their similarity to fungal viruses suggests transmission across kingdoms. Plants are almost always colonized by numerous endophytic fungi and, during this interaction, an opportunity for virus exchange between plant and fungal cells exists. Until now, persistent plant and fungal viruses have been recognized as occurring only in the families *Partitiviridae* and *Endornaviridae* [38], and phylogenetic analyses of these families support the transmission of these viruses among plant and fungal hosts [37]. A persistent virus discovered in blueberry does not appear to be a member of these families and, interestingly, has a genome organization related to members of the family *Totiviridae* [39].

Most persistent plant viruses have dsRNA genomes and encode only an RdRp and a CP, lacking the movement protein crucial to systemic infection. Therefore, there is no cell-to-cell movement or transport of the virus within the plant except when cell division takes place [40]. Although the PMeV genome does not encode a movement protein, the virus can infect papaya plants systemically. As PMeV is located only in the laticiferous vessels fo the plant [7], the increased latex fluidity and exudation during the infection suggest that the virus uses the laticiferous vessels to move systemically throughout the plant. However, a different scenario may occur during mixed infection: PMeV could use a movement protein from PMeV2. The ORF3 protein encoded by umbraviruses plays an essential role in mediating long-distance movement [35]. Although PMeV2 does not have a protein with similarity with the ORF3 protein, the putative protein encoded by ORF1 could be responsible for virus movement.

PMeV is an unusual plant virus: it is localized on and linked to the polymers present in the latex, a completely hostile environment for viral replication; it is the first plant virus related to viruses in the family *Totiviridae*; it acts like a persistent virus in single infections; and its CP encapsidates the ssRNA genome of the umbravirus-like PMeV2.

Our results indicate that papaya sticky disease is associated with a combination of PMeV, a toti-like virus, and PMeV2, an umbra-like virus. Considering the importance of the papaya industry in several tropical countries, a more accurate diagnostic method allowing the detection of both PMeV and PMeV2 is urgently necessary for the early detection of these agents with the purpose of reducing economic losses and preventing the introduction of these two viruses into new areas.

## Supporting Information

S1 Fig RNA banding pattern (0.8% agarose gel) obtained from the latex of asymptomatic and symptomatic papaya plants. 1, RNA band corresponding to the papaya meleira virus (PMeV) genome. 2, RNA band corresponding to the papaya meleira virus 2 (PMeV2) genome. S1, S2 and S4 are different symptomatic plants, and S3 is an asymptomatic plant. M, size marker [1 kb plus DNA ladder (Invitrogen), in kbp].
(TIF)

S1 Table List of primers used for virus detection and re-amplification of PMeV and PMeV2 genomes. (DOC)

S2 Table Names, acronyms, accession numbers and taxonomic position of the viruses included in the phylogenetic analysis. (PDF)

## References

[1] Rodrigues CH, Ventura JA, Maffia LA. Distribuição e transmissão da meleira em pomares de mamão no Espírito Santo. Fitopatologia Brasileira 1989;14:118.

[2] Daltro CB, Abreu EFM, Aragão FJL, Andrade EC. Genetic diversity studies of Papaya meleira virus. Tropical Plant Pathology. 2014;39(1):104–8.

[3] Perez-Brito D, Tapia-Tussell R, Cortes-Velazquez A, Quijano-Ramayo A, Nexticapan-Garcez A, Martín-Mex R. First report of *Papaya meleira virus* (PMeV) in Mexico. African Journal of Biotechnology. 2012;11(71):13564–70.

[4] Abreu P, Antunes TF, Magaña-Álvarez A, Pérez-Brito D, Tapia-Tussell R, Ventura JA, et al A Current Overview of the Papaya meleira virus, an Unusual Plant Virus. Viruses. 2015;7(4):1853–70. 10.3390/v7041853 25856636 PMC4411680

[5] Ventura JA, Costa H, da Silva Tatagiba J. Papaya diseases and integrated control Diseases of Fruits and Vegetables: Volume II: Springer; 2004 p. 201–68.

[6] Rodrigues SP, Da Cunha M, Ventura JA, Fernandes PMB. Effects of the Papaya meleira virus on papaya latex structure and composition. Plant cell reports. 2009;28(5):861–71. 10.1007/s00299-009-0673-7 19194708

[7] Kitajima EW, Rodrigues CH, Silveira JS, Alves F, Ventura JA, Aragao FJL, et al Association of isometric viruslike particles, restricted to laticifers, with "meleira" ("Sticky disease") of papaya (Carica papaya). Fitopatologia Brasileira. 1993;18:118–22.

[8] Maciel-Zambolim E, Kunieda‐Alonso S, Matsuoka K, De Carvalho M, Zerbini F. Purification and some properties of Papaya meleira virus, a novel virus infecting papayas in Brazil. Plant Pathology. 2003;52(3):389–94.

[9] Araújo MMMd, Tavares ÉT, Silva FRd, Marinho VLdA, Júnior MTS. Molecular detection of *Papaya meleira virus* in the latex of *Carica papaya* by RT-PCR. Journal of virological methods. 2007;146(1):305–10. 17826848 10.1016/j.jviromet.2007.07.022

[10] Abreu EF, Daltro CB, Nogueira EO, Andrade EC, Aragao FJ. Sequence and genome organization of papaya meleira virus infecting papaya in Brazil. Archives of Virology. 2015;(160):3143–7. 10.1007/s00705-015-2605-x 26370790

[11] Tapia-Tussell R, Magaña‐Alvarez A, Cortes-Velazquez A, Itza-Kuk G, Nexticapan-Garcez A, Quijano-Ramayo A, et al Seed transmission of Papaya meleira virus in papaya (Carica papaya) cv. Maradol. Plant Pathology. 2015;64(2):272–5.

[12] Zamudio-Moreno E, Ramirez-Prado J, Moreno-Valenzuela O, Lopez-Ochoa L. Early diagnosis of a Mexican variant of Papaya meleira virus (PMeV-Mx) by RT-PCR. Genetics and Molecular Research. 2015;14(1):1145–54. 10.4238/2015.February.6.18 25730054

[13] Rodrigues SP, Galvao OP, Andrade JS, Ventura JA, Fernandes PMB. Simplified molecular method for the diagnosis of Papaya meleira virus in papaya latex and tissues. Summa Phytopathologica. 2005;31:281–3.

[14] Sperschneider J, Datta A. DotKnot: pseudoknot prediction using the probability dot plot under a refined energy model. Nucleic acids research. 2010;38(7):e103–e. 10.1093/nar/gkq021 20123730 PMC2853144

[15] Edgar RC. MUSCLE: multiple sequence alignment with high accuracy and high throughput. Nucleic acids research. 2004;32(5):1792–7. 15034147 10.1093/nar/gkh340 PMC390337

[16] Ronquist F, Huelsenbeck JP. MrBayes 3: Bayesian phylogenetic inference under mixed models. Bioinformatics. 2003;19(12):1572–4. 12912839 10.1093/bioinformatics/btg180

[17] Shevchenko A, Tomas H, Havli J, Olsen JV, Mann M. In-gel digestion for mass spectrometric characterization of proteins and proteomes. Nature protocols. 2006;1(6):2856–60. 17406544 10.1038/nprot.2006.468

[18] Keller A, Nesvizhskii AI, Kolker E, Aebersold R. Empirical statistical model to estimate the accuracy of peptide identifications made by MS/MS and database search. Analytical chemistry. 2002;74(20):5383–92. 12403597 10.1021/ac025747h

[19] Nesvizhskii AI, Keller A, Kolker E, Aebersold R. A statistical model for identifying proteins by tandem mass spectrometry. Analytical chemistry. 2003;75(17):4646–58. 14632076 10.1021/ac0341261

[20] Blom N, Hansen J, Brunak S, Blaas D. Cleavage site analysis in picornaviral polyproteins: discovering cellular targets by neural networks. Protein Science. 1996;5(11):2203–16. 8931139 10.1002/pro.5560051107 PMC2143287

[21] Livshits M, Amosova O, Lyubchenko YL. Flexibility difference between double-stranded RNA and DNA as revealed by gel electrophoresis. Journal of Biomolecular Structure and Dynamics. 1990;7(6):1237–49. 1973043 10.1080/07391102.1990.10508562

[22] Cai G, Krychiw JF, Myers K, Fry WE, Hillman BI. A new virus from the plant pathogenic oomycete Phytophthora infestans with an 8 kb dsRNA genome: the sixth member of a proposed new virus genus. Virology. 2013;435(2):341–9. 10.1016/j.virol.2012.10.012 23146209

[23] Kozlakidis Z, Hacker CV, Bradley D, Jamal A, Phoon X, Webber J, et al Molecular characterisation of two novel double-stranded RNA elements from Phlebiopsis gigantea. Virus genes. 2009;39(1):132–6. 10.1007/s11262-009-0364-z 19430898

[24] Alam SL, Atkins JF, Gesteland RF. Programmed ribosomal frameshifting: Much ado about knotting! Proceedings of the National Academy of Sciences. 1999;96(25):14177–9. 10.1073/pnas.96.25.14177 PMC33937 10588670

[25] Yu L, Sang W, Wu M-D, Zhang J, Yang L, Zhou Y-J, et al Novel Hypovirulence-Associated RNA Mycovirus in the Plant-Pathogenic Fungus Botrytis cinerea: Molecular and Biological Characterization. Applied and environmental microbiology. 2015;81(7):2299–310. 10.1128/AEM.03992-14 25595766 PMC4357927

[26] Brierley I, Pennell S, Gilbert RJ. Viral RNA pseudoknots: versatile motifs in gene expression and replication. Nature Reviews Microbiology. 2007;5(8):598–610. 17632571 10.1038/nrmicro1704 PMC7096944

[27] Ryabov E, Taliansky M, Robinson DG, Waterhouse P, de Zoeten G, Falk B, et al Genus Umbravirus. Virus Taxonomy: Ninth Report of the International Committee on Taxonomy of Viruses Virus Taxonomy. 2012:1191–5.

[28] Speir JA, Johnson JE. Virus particle structure: non-enveloped viruses In: Mahy BWJ VR M, editor. Encyclopedia of Virology. 3 ed. Oxford, UK: Elsevier Academic Press; 2008 p. 380–93.

[29] Fauquet C, Fargette D. International Committee on Taxonomy of Viruses and the 3,142 unassigned species. Virol J. 2005;2:64 16105179 10.1186/1743-422X-2-64 PMC1208960

[30] Roossinck MJ, Saha P, Wiley GB, Quan J, White JD, Lai H, et al Ecogenomics: using massively parallel pyrosequencing to understand virus ecology. Molecular Ecology. 2010;19(s1):81–8. 20331772 10.1111/j.1365-294X.2009.04470.x

[31] Liu H, Fu Y, Xie J, Cheng J, Ghabrial SA, Li G, et al Discovery of novel dsRNA viral sequences by in silico cloning and implications for viral diversity, host range and evolution. PloS one. 2012;7(7). 10.1371/journal.pone.0042147 PMC3407116 22848734

[32] Chen S, Cao L, Huang Q, Qian Y, Zhou X. The complete genome sequence of a novel maize-associated totivirus. Archives of virology. 2015:1–4. 10.1007/s00705-015-2657-y 26559960

[33] Kreuze J. siRNA deep sequencing and assembly: piecing together viral infections Detection and Diagnostics of Plant Pathogens: Springer; 2014 p. 21–38.

[34] Quito-Avila D, Alvarez R, Ibarra M, Martin R. Detection and partial genome sequence of a new umbra-like virus of papaya discovered in Ecuador. European Journal of Plant Pathology. 2015:1–6.

[35] Taliansky ME, Robinson DJ. Molecular biology of umbraviruses: phantom warriors. Journal of General Virology. 2003;84(8):1951–60. 12867625 10.1099/vir.0.19219-0

[36] Abraham A, Menzel W, Varrelmann M, Vetten HJ. Molecular, serological and biological variation among chickpea chlorotic stunt virus isolates from five countries of North Africa and West Asia. Archives of virology. 2009;154(5):791–9. 10.1007/s00705-009-0374-0 19347243 PMC3085786

[37] Roossinck MJ. Plant virus ecology. 2013. 10.1371/journal.ppat.1003304 PMC3662651 23717199

[38] King AM, Adams MJ, Lefkowitz EJ. Virus taxonomy: classification and nomenclature of viruses: Ninth Report of the International Committee on Taxonomy of Viruses: Elsevier; 2011.

[39] Martin RR, Zhou J, Tzanetakis IE. Blueberry latent virus: an amalgam of the Partitiviridae and Totiviridae. Virus research. 2011;155(1):175–80. 10.1016/j.virusres.2010.09.020 20888379

[40] Boccardo G, Lisa V, Luisoni E, Milne RG. Cryptic plant viruses. Adv Virus Res. 1987;32:171–214. 3303860 10.1016/s0065-3527(08)60477-7

