# Supplementary figures and images for: The dsRNA Virus Papaya Meleira Virus and an ssRNA Virus Are Associated with Papaya Sticky Disease
Source: PLoS One. 2016 May 11;11(5):e0155240. doi: 10.1371/journal.pone.0155240 (PMC4863961; doi:10.1371/journal.pone.0155240)

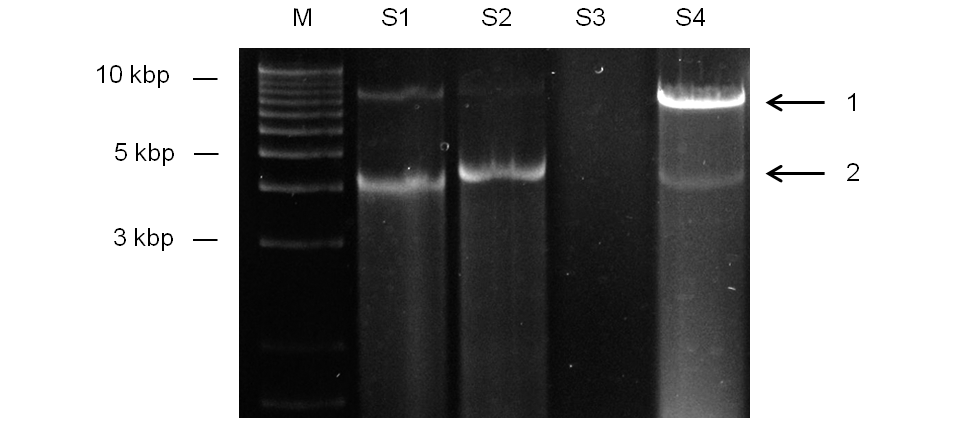

Supplement: S1 Fig — 1, RNA band corresponding to the papaya meleira virus (PMeV) genome. 2, RNA band corresponding to the papaya meleira virus 2 (PMeV2) genome. S1, S2 and S4 are different symptomatic plants, and S3 is an asymptomatic plant. M, size marker [1 kb plus DNA ladder (Invitrogen), in kbp]. (TIF) [file pone.0155240.s001.tif]
