# Supplementary material for: The dsRNA Virus Papaya Meleira Virus and an ssRNA Virus Are Associated with Papaya Sticky Disease
Source: PLoS One. 2016 May 11;11(5):e0155240. doi: 10.1371/journal.pone.0155240 (PMC4863961; doi:10.1371/journal.pone.0155240)
Supplement: S1 Table — (DOC) [file pone.0155240.s002.doc]

| **Amplicon** | **Primer pair** | **Sequences** | **Tm*** | **AmpliconSize** |
| --- | --- | --- | --- | --- |
| A1 | PMeV1.1 | F: 5´ GGGCGTTGACCTGGATATG 3´  R: 5´ AGCCTGAGCCCATCTAGTAA 3´ | 55 °C | 2008 |
| A2 | PMeV1.2 | F: 5´ GCTTTCCAGATTCATCGTTGTT 3´  R: 5´ GGTCCTACTCTTTGAGCATCAG 3´ | 55 °C | 2204 |
| A3 | PMeV1.3 | F: 5´ CACCTAGTATGGCAACGAATCA 3´  R: 5´ CCTAAAGGACTACTAATCGG 3´ | 55 °C | 2161 |
| A4 | PMeV1.4 | F: 5´ GCTTGGGTGGTAAGACACAT 3´  R: 5´ CAGTGCTAATAACCTGATGT 3´ | 55 °C | 2031 |
| A5 | PMeV1.5 | F: 5´ CTGATGTTAGGGCAGGATGTT 3´  R: 5´ ACAAGGAGGAAATAGGAGAA 3´ | 55 °C | 1052 |
| A6 | PMeV1.6 | F: 5´ CACTGACCAGTTACTTA 3´  R: 5´ CTTGATCCGTTACTAGA 3´ | 50 °C | 1073 |
| A7 | PMeV1.7 | F: 5´ GCTCCTCCATCTTTCTT 3´  R: 5´ GGAACCTTTGATACCTT 3´ | 53 °C | 1038 |
| A8 | PMeV2.1 | F: 5´ GTGAATTATGTTGGCATACAACGAG 3´  R: 5´ GGTAGATGGTATACACACAATACAG 3´ | 62 °C | 1701 |
| A9 | PMeV2.2 | F: 5´ CAAGTGGGATAAGTTCAGAGA 3´  R: 5´ CAACTCTCAAGCCTTTAGAT 3´ | 58 °C | 1734 |
| A10 | PMeV2.3 | F: 5´ ATTCAAGTAGTGGAGCAGGCT 3´  R: 5´ CTGCGACTTGGTACTTAAAGG 3´ | 58°C | 1697 |
| A11 | PMeV | F: 5´ CTTGGTTAGGCATAACTGTAGGT 3´  R: 5´ CACGGACTCTTAGAAACGTCTATC 3´ | 60 °C | 394 |
| A12 | PMeV2 | F: 5´ CCAATGCCCATAAAGATAGCACT 3´  R: 5´ CTGAAATCGGAGGTCTTGTCATA 3´ | 60 °C | 754 |
| A13 | Luteovirus | F: 5' GCTCTAGAATTGTTAATGARTACGGTCG 3'  R 5'CACGCGTCIACCTATTTIGGRTTITG3' | 57° | 850 |
