# Supplementary material for: The dsRNA Virus Papaya Meleira Virus and an ssRNA Virus Are Associated with Papaya Sticky Disease
Source: PLoS One. 2016 May 11;11(5):e0155240. doi: 10.1371/journal.pone.0155240 (PMC4863961; doi:10.1371/journal.pone.0155240)
Supplement: S2 Table — (PDF) [file pone.0155240.s003.pdf]

**Virus name**	**Acronym**	**Accession number**	**Taxonomic position (family/genus)**
Black raspberry virus F	BRV-F	NC_009890	*Totiviridae*/unassigned
Botryotinia fuckeliana totivirus	BfV	AM491608	*Totiviridae*/unassigned
Botrytis cinerea RNA virus 1	BcRV-1	NC_026139	Tentative *Totiviridae*
Coniothyrium minitans RNA virus	CmRV	NC_007523	*Totiviridae*/*Victorivirus*
Diplodia scrobiculata RNA virus 1	DsRV-1	NC_013699	Tentative *Totiviridae*
Eimeria brunetti RNA virus 1	EbRV-1	NC_002701	Tentative *Totiviridae*
Fusarium graminearum dsRNA mycovirus-3	FgV-3	NC_013469	Tentative *Totiviridae*
Fusarium virguliforme dsRNA mycovirus 1	FvRV-1	JN671444	Tentative *Totiviridae*
Fusarium virguliforme dsRNA mycovirus 2	FvRV-2	JN671443	Tentative *Totiviridae*
Giardia lamblia virus	GLV	NC_003555	*Totiviridae*/*Giardiavirus*
Gremmeniella abietina RNA virus L1	GaV-L1	AF337175	*Totiviridae*/*Victorivirus*
Gremmeniella abietina RNA virus L2	GaV-L2	NC_005965	*Totiviridae*/unassigned
Leishmania RNA virus 1-1	LRV1-1	NC_002063	*Totiviridae*/*Leishmaniavirus*
Leishmania RNA virus 2-1	LRV2-1	NC_002064	*Totiviridae*/*Leishmaniavirus*
Phlebiopsis gigantea dsRNA 1	PgV-1	NC_013999	Tentative *Totiviridae*
Phlebiopsis gigantea dsRNA 2	PgV-2	AM111097	Tentative *Totiviridae*
Phytophthora infestans RNA virus 3	PiRV-3	JN603241	Tentative *Totiviridae*
Saccharomyces cerevisiae virus L-A	ScVL-A	NC_003745	*Totiviridae*/*Totivirus*
Sclerotinia sclerotiorum dsRNA mycovirus-L	SsNsV-L	NC_017915	Tentative *Totiviridae*
Sphaeropsis sapinea RNA virus 2	SsRV-2	NP_047560.1	*Totiviridae*/*Victorivirus*
Tuber aestivum virus 1	TaV-1	HQ158596	*Totiviridae*/*Totivirus*
Trichomonas vaginalis virus 1-1	TVV-1	JF436869	*Totiviridae*/*Trichomonasvirus*
Trichomonas vaginalis virus 2	TVV-2	HQ607514	*Totiviridae*/*Trichomonasvirus*
Ustilago maydis virus H1	UmV-H1	NC_003823	*Totiviridae*/*Totivirus*
Beet black scorch virus	BBSV	JN635328	*Tombusviridae/Betanecrovirus*
Carrot mottle virus	CMoV	NC_011515	*Tombusviridae/Umbravirus*
Carrot mottle mimic virus	CMoMV	NC_001726	*Tombusviridae/Umbravirus*
citrus yellow vein-associated virus	CYVaV	JX101610	Unassigned
Cowpea mottle virus	CPMoV	NC_003535	*Tombusviridae/Carmovirus*
Galinsoga mosaic virus	GaMV	NC_001818	*Tombusviridae/Gallantivirus*
Groundnut rosette virus	GRV	NC_003603	*Tombusviridae/Umbravirus*
Maize chlorotic mottle virus	MCMV	KF010583	*Tombusviridae/Machlomovirus*
Melon necrotic spot virus	MNSV	JX879088	*Tombusviridae/Carmovirus*
Olive mild mosaic virus	OMMV	AY616760	*Tombusviridae/Alphanecrovirus*
Olive latent virus 1	OLV-1	NC_001721	*Tombusviridae/Alphanecrovirus*
Opium poppy mosaic virus	OpPMV	NC_027710	*Tombusviridae*/ unassigned
papaya meleira virus-Mx	PMeV-Mx	KF214786	*Tombusviridae*/ unassigned
papaya virus Q	PpVQ	KP165407	*Tombusviridae*/ unassigned
Pea enation mosaic virus-2	PEMV-2	NC_003853	*Tombusviridae/Umbravirus*
Tobacco bushy top virus	TBTV	FM242700	*Tombusviridae/Umbravirus*
Tobacco necrosis virus A	TNV-A	GQ221829	*Tombusviridae/Alphanecrovirus*
Tobacco necrosis virus D	TNV-D	NC_003487	*Tombusviridae/Betanecrovirus*
Turnip crinkle virus	TCV	NC_003821	*Tombusviridae/Carmovirus*
